# Value of Echocardiography and Cardiac Magnetic resonance in assessing left ventricular function in breast and gastric cancer patients after Anthracycline Chemotherapy

**DOI:** 10.1186/s12872-023-03495-2

**Published:** 2023-09-15

**Authors:** Chao-long Jin, Xue-gong Shi, Ting-ting Wang, Hong-wen Li, Ding-Xin Zhang, Zhe Sheng, Jie Xiao, Yong-Qiang Yu

**Affiliations:** 1 Cardiac Imaging Center, The First Affiliated Hospital of Anhui Medical University, Hefei, Anhui 230022 China; 2 MRI Room, The First Affiliated Hospital of Anhui Medical University, Hefei, Anhui 230022 China; 3 Department of Radiology, The First Affiliated Hospital of Anhui Medical University, Hefei, Anhui 230022 China

**Keywords:** Chemotherapy, Cardiac magnetic resonance imaging, Echocardiography, Left ventricular function, Cardiotoxicity

## Abstract

**Background:**

Echocardiography (ECHO) and cardiac magnetic resonance imaging (MRI) are used to observe changes in the left ventricular structure in patients with breast and gastric cancer after 6 cycles of chemotherapy. Based on the observed values, we aimed to evaluate the cardiotoxicity of anthracyclines in cancer patients and to analyze the consistency of the two examination methods in assessing left ventricular function after chemotherapy.

**Methods:**

From January 2020 to January 2022, the data of 80 patients with malignant tumors who received anthracycline chemotherapy (breast cancer, n = 40; gastric cancer, n = 40) and 40 healthy volunteers (Control group) were retrospectively collected. Serum high-sensitivity cardiac troponin T (hs-cTnT) levels were detected by an automatic immunoassay analyzer. Left ventricular end-systolic volume (LVESV), left ventricular end-diastolic volume (LVEDV) and left ventricular ejection fraction (LVEF) were measured by cardiac MRI and 2-dimensional ECHO using the biplane Simpson’s method.

**Results:**

Compared with baseline values, serum high-sensitivity cardiac troponin T (hs-cTnT) levels were significantly increased in patients with breast cancer and gastric cancer after 6 cycles of chemotherapy (*P* < 0.05). In addition, LVEDV, LVESV and LVEF measured with MRI were higher than those detected by ECHO in cancer patients after 6 cycles of chemotherapy (*P* < 0.05). And the Bland-Altman plot analysis showed that LVEDV, LVESV and LVEF measured by the two examination methods were in good agreement.

**Conclusion:**

Breast and gastric cancer patients exhibited elevated levels of hs-cTnT after 6 cycles of chemotherapy, indicating potential cardiotoxicity. Additionally, cardiac MRI and 2-dimensional ECHO showed good agreement in assessing left ventricular function, with ECHO tending to underestimate volume measurements compared to MRI.

## Background

Tumors are the most common malignant disease and one of the leading causes of death worldwide [1]. In the past decades, the survival rate of cancer patients has been greatly improved due to the early detection of cancer and the advent of novel treatment modalities [2]. Chemotherapy is a widely used treatment regimen [3], and anthracyclines (ANT) are common medications for chemotherapy. However, ANT is associated with several adverse events, of which cardiotoxicity is the most serious [4, 5]. It has previously been reported that the incidence of ANT-induced heart failure increased with increasing cumulative dosage; for instance, the incidence was 3–5% at 400 mg/m^2^ and could reach as high as 18–48% for ANT at 700 mg/m^2^ [6]. In addition, ANT-related toxicity after long-term chemotherapy can increase the morbidity and mortality of cancer survivors [7].

Cardiotoxicity leads to a decrease in the left ventricular ejection fraction (LVEF) or an increase in serum troponin in patients due to myocardial dysfunction, seriously affecting the prognosis and quality of life of patients after chemotherapy [8]. Depending on the medication, the time to onset of cardiotoxicity varies [9]. At present, the prevalent techniques to detect cardiotoxicity are tests to evaluate left ventricular function (LVEF measurement and speckle tracking imaging) [10]. Echocardiography (ECHO), which can evaluate subendocardial left ventricular longitudinal strain, is an early detection method for ANT-induced left ventricular myocardial dysfunction [9, 11]. It is widely used due to its noninvasive methods, quick and reliable results, and cost-effectiveness [12]. However, it should be noted that ECHO might be limited in terms of poor intraobserver and interobserver reproducibility and accuracy [13]. Liu et al. [14] compared the results of left ventricular function by ECHO and cardiac magnetic resonance imaging (MRI) in hypertensive patients with ventricular hypertrophy and found that the left ventricular end-diastolic volume (LVEDV), left ventricular end-systolic volume (LVESV), stroke volume and LVEF measured by the two examination methods were in good agreement. The advantages of cardiac MRI are that the images generated are remarkably complete, detailed, and precise, and to a certain extent, better than other cardiac imaging tests, and most importantly, its associated low inter-reader variability compared to two-dimensional for left ventricular function and volumes but might not be commonly used due to its high cost and complex maintenance, especially in less-resourced institutions [15]. Currently, there are few studies on the consistency of ECHO and cardiac MRI in assessing left ventricular function after anthracycline chemotherapy in cancer patients.

Therefore, in this study, we investigated the effects of chemotherapy on the ventricular function in gastric and breast cancer patients using ECHO and cardiac MRI and compared the differences between the two examination methods. Overall, this study aimed to promote the early and accurate evaluation of cardiac function for the comprehensive treatment of patients, minimize cardiovascular adverse events, and improve prognosis.

## Materials and methods

### General information of patients

**Fig. 1:**
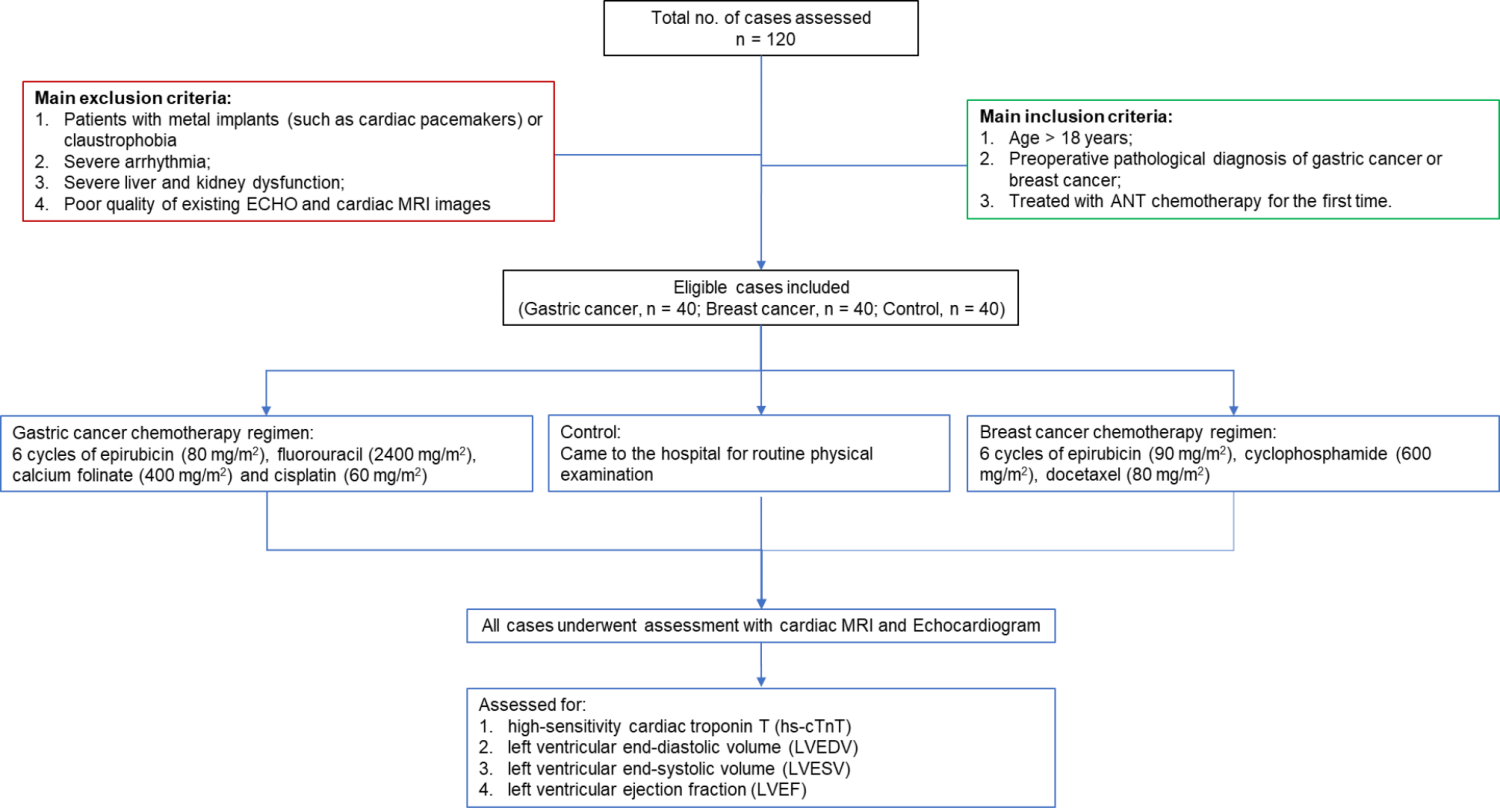
Flow chart of case selection

Data from 80 patients diagnosed with malignant tumors and treated with ANT chemotherapy (Cancer group) and 40 volunteers who came to our hospital for health examinations (Control group) at our hospital from January 2020 to January 2022 were collected and retrospectively analyzed. The 80 patients in the Cancer group included 40 patients with breast cancer and 40 patients with gastric cancer. The study inclusion criteria were: (1) age > 18 years; (2) preoperative pathological diagnosis of gastric cancer or breast cancer; (3) treated with ANT chemotherapy for the first time. Exclusion criteria were: (1) patients with metal implants (such as cardiac pacemakers) or claustrophobia; (2) severe arrhythmia; (3) severe liver and kidney dysfunction; (4) poor quality of existing ECHO and cardiac MRI images. The flow chart of case selection is shown in Fig. 1.

The following data of patients were recorded: age, body mass index, blood pressure, past medical history, tumor stage, serum high-sensitivity cardiac troponin T (hs-cTnT), ECHO and cardiac MRI measures. Then, the general baseline data and left ventricular function-related indicators in the Cancer group before chemotherapy were compared with Control groups. Additionally, assessment results of left ventricular function by ECHO and cardiac MRI after 6 cycles of chemotherapy were compared, and we further evaluated the agreement between ECHO and cardiac MRI in the measurement of left ventricular function. Informed consent was obtained from all patients and healthy individuals. The study protocol was reviewed and approved by the Ethics Committee of The First Affiliated Hospital of Anhui Medical University (PJ2020-07-23).

### Chemotherapy regimens

Patients with gastric cancer were treated with a combined regimen including epirubicin, fluorouracil, calcium folinate, and cisplatin for 6 cycles (Chemotherapy was administered for each 3-week cycle consisting of epirubicin (80 mg/m^2^), fluorouracil (2400 mg/m^2^), calcium folinate (400 mg/m^2^) and cisplatin (60 mg/m^2^) via intravenous infusion on day 1, and fluorouracil 400 mg/m^2^ intravenous injection first, then 2400 mg/m^2^ by continuous intravenous infusion for 46 h). Breast cancer patients were treated with a combined regimen of epirubicin and cyclophosphamide for 6 cycles (Chemotherapy was administered for each 3-week cycle consisting of epirubicin (90 mg/m^2^), cyclophosphamide (600 mg/m^2^) and docetaxel (80 mg/m^2^) via intravenous infusion).

### Test methods

#### Cardiac magnetic resonance imaging

Cardiac MRI scanning was performed using a 3.0 T Siemens Prisma MRI scanner (Siemens, Germany) with a standard steady-state free precession breath-hold sequence. The specific scanning parameters were: FOV: 350 mm × 350 mm, matrix: 176 × 150, TR: 3.1 ms, TE: 1.53 ms, flip angle: 60°, short-axis slice thickness of 8.0 mm, and acquisition slices: 8 to 12. Three long-axis images and short-axis images covering the entire left ventricle were obtained. CVI42 software (Circle Cardiovascular Imaging, Calgary, Canada) was used to determine LVESV, LVEDV and LVEF from the MR images. Papillary muscles were included. AI automatically tracks Endocardium. If Endocardium tracking is good, volume can be measured automatically directly. If Endocardium tracking is bad, volume can be measured after manual adjustment.

#### Echocardiogram

The patient was placed in the left lateral decubitus position, and the ECG was connected. The GE Vivid E95 Color Doppler ultrasound machine was equipped with a two-dimensional probe M5Sc-D (frequency of 1.5 to 4.6 MHz) and EchoPAC software was selected to obtain the standard AP4 and AP2 sections. The ED and ES phases were determined when the apex of the heart was clearly visible and the left ventricular long axis was perpendicular to the mitral ring plane. Then, the left ventricular endocardial trace of AP4 and AP2 was recorded manually, starting from the mitral septum at the side valve ring and ending at the side valve ring to obtain the left ventricular LVEDV and LVESV volumes, and LVEF was calculated automatically.

#### Determination of serum high-sensitivity cardiac troponin T (hs-cTnT) level

Venous blood was collected from patients in the morning under a fasting state. Using the VIDAS automatic Fluorescence immunity analyzer (French BioMerieux company), their serum high-sensitivity cardiac troponin T (hs-cTnT) level was measured in strict accordance with the instructions of the electrochemiluminescence immunoassay kit.

### Statistical analysis

SPSS 22.0 was used for data processing. Measurement data are expressed as mean ± standard deviation (SD). The t-test was used for comparison between two groups, and one-way analysis of variance was used for comparison between multiple groups with normal distribution, Bonferroni was used if accord with homogeneity of variances, Dunnett’s T3 was used if not accord. Non-normal distribution data were described using median (Q25, Q75) and compared with non-parametric rank sum tests. The Z-test was employed for the rank sum test of the two groups, while the K-W test (H test) was used for the rank sum test of multiple groups. Enumeration data were expressed as number or percent and compared using the χ^2^ test. The Bland-Altman plot was utilized for consistency analysis. *P* < 0.05 was considered statistically significant.

## Results

### Baseline characteristics

A total of 120 subjects were enrolled in this study, including 40 patients with breast cancer [median age, 55 (43,65)], 40 patients with gastric cancer [median age,53 (44,61)] and 40 healthy subjects [median age, 54 (47,63)]. Before chemotherapy, there was no significant difference among them in age, body mass index, systolic blood pressure, diastolic blood pressure, heart rate, history of hypertension, history of cardiovascular disease, tumor stage, serum hs-cTnT, LVESV, LVEDV and LVEF (*P* > 0.05) (Table 1).

**Table 1:** Comparison of baseline data among groups

Variable	Control group (n = 40)	Cancer group		*χ2/t/F/H*	*P*
		Breast cancer (n = 40)	Gastric cancer (n = 40)		
Age	54 (47, 63)	55 (43, 65)	53 (44, 61)	0.598	0.615
Body mass index	27.02 ± 2.03	27.60 ± 1.62	27.38 ± 1.35	2.035	0.133
Systolic blood pressure	135.55 ± 17.37	133.22 ± 19.46	128.15 ± 18.64	2.503	0.084
Diastolic blood pressure	90.97 ± 18.38	92.08 ± 15.58	87.73 ± 10.58	1.434	0.241
Heart rate	82.82 ± 8.89	83.15 ± 6.84	84.10 ± 7.87	0.440	0.645
History of hypertension (Yes/No)	18/22	17/23	13/27	1.458	0.482
History of cardiovascular disease (Yes/No)	10/30	9/31	9/31	0.093	0.954
Tumor stage (1/2/3/4)	-	13/21/4/2	13/17/7/3	1.452	0.693
8th AJCC TNM classification	-			1.452	0.693
I	-	13	13		
II	-	21	17		
III	-	4	7		
IV	-	2	3		
Serum hs-cTnT (ng/L)	8 (2.5, 16.75)	7 (2, 15)	8 (2, 14)	0.118	0.943
LVEDV-MRI (ml)	106.93 ± 15.16	110.94 ± 16.02	111.86 ± 16.32	0.265	0.767
LVESV-MRI (ml)	42.54 ± 8.06	45.54 ± 10.07	44.12 ± 9.17	0.857	0.427
LVEF-MRI (%)	60.31 ± 4.86	58.99 ± 6.34	60.66 ± 4.63	1.149	0.320
LVEDV- Echo (ml)	86.98 ± 15.57	91.15 ± 10.49	90.25 ± 13.09	0.089	0.915
LVESV-Echo (ml)	37.87 ± 10.23	40.69 ± 5.52	38.59 ± 7.53	0.888	0.414
LVEF-Echo (%)	56.65 ± 7.99	55.33 ± 3.79	57.26 ± 5.15	1.406	0.249

Note: hs-cTnT, high-sensitivity cardiac troponin T (hs-cTnT); LVEDV, left ventricular end-diastolic volume; LVESV, left ventricular end-systolic volume; LVEF, left ventricular ejection fraction

### Comparison of serum high-sensitivity cardiac troponin T (hs-cTnT) Level before and after chemotherapy

Serum hs-cTnT level in patients with gastric cancer after chemotherapy [9.3 (3.4–882.1) pg/mL] was increased compared with that before chemotherapy [8 (2–14) pg/mL], and the difference was statistically significant (*P* < 0.001) (Table 2). Serum hs-cTnT level in patients with breast cancer after chemotherapy [13.5 (4.1–843.9) pg/mL] was increased compared with that before chemotherapy [7 (2,15) pg/mL], and the difference was statistically significant (*P* < 0.001) (Table 2).

**Table 2:** Comparison of serum high-sensitivity troponin T (hs-cTnT) levels at different time points in cancer patients

Cancer type	Before chemotherapy	After 6 cycles of chemotherapy	*Z*	*P*
Gastric cancer (n = 40)	8 (2, 14)	9.3 (3.4, 882.1)	-3.509	< 0.001
Breast cancer (n = 40)	7 (2, 15)	13.5 (4.1, 843.9)	-3.622	< 0.001

### Consistency analysis of cardiac function indexes by Echocardiogram and cardiac magnetic resonance imaging in cancer patients

**Fig. 2:**
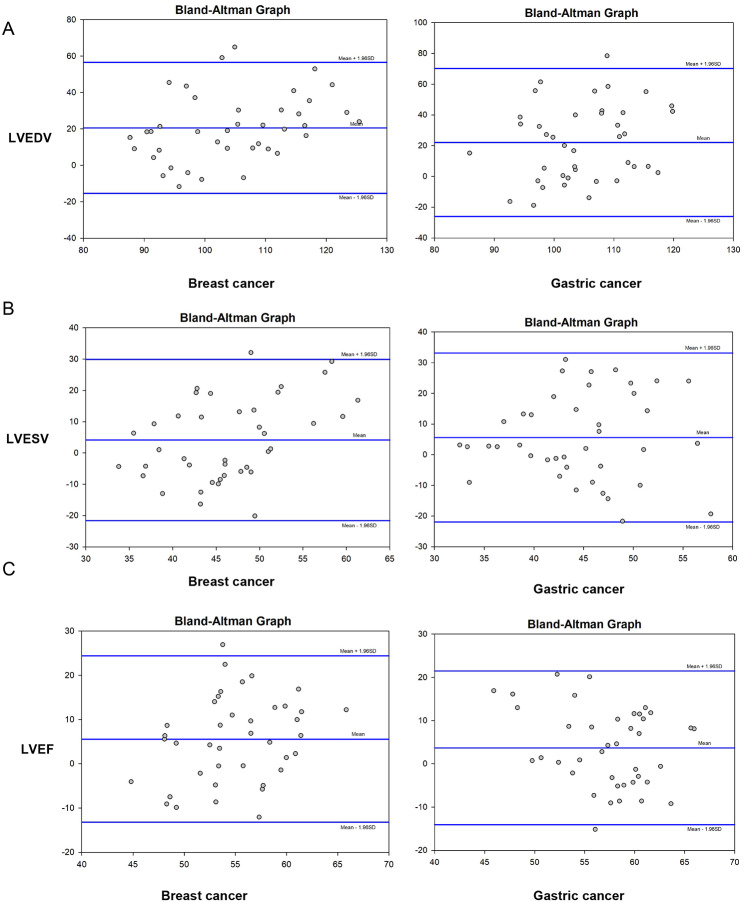
Bland-Altman plot for consistency analysis of LVEDV, LVESV and LVEF measurements from echocardiogram and cardiac magnetic resonance in patients with breast cancer and gastric cancer after 6 cycles of chemotherapy. Bland-Altman plots showing differences (delta) in measurements of LVEDV (**A**), LVESV (**B**), LVEF (**C**) for breast and gastric cancer. The middle lines represent the mean (2 SD) of the difference, and the upperlines and lowerlines represent the upperlimit and lowerlimit of the difference, respectively

After 6 cycles of chemotherapy, LVEDV, LVESV and LVEF of cancer patients measured with MRI were higher than those measured with ECHO (*P* < 0.05) (Table 3). And the same result was also for the control group. Meanwhile, Bland-Altman plots analysis showed large differences in LVEDV, LVESV and LVEF values measured by ECHO and cardiac MRI in patients with breast cancer and gastric cancer. Limits of agreement expressed as mean difference between ECHO and cardiac MRI measurements were 20.6 in breast cancer and 22.1 in gastric cancer for LVEDV, 4.2 in breast cancer and 5.6 in gastric cancer for LVESV; 5.6 in breast cancer and 3.7 in gastric cancer for LVEF. As most spots are within the upperlimits and lowerlimts, which showed that LVEDV, LVESV and LVEF measured by the two examination methods were in good agreement (Fig. 2).

**Table 3:** Comparison of cardiac function indexes between the two groups after 6 cycles of chemotherapy

Assessment indexes		Control group (n = 40)	Breast cancer (n = 40)	Gastric cancer (n = 40)
LVEDV (ml)	MRI	106.93 ± 15.16	114.28 ± 16.15	117.00 ± 16.36
	Echo	86.98 ± 15.57	93.21 ± 11.27	94.78 ± 13.07
t		5.805	6.765	6.446
*P*		0.000	0.000	0.000
LVESV (ml)	MRI	42.54 ± 8.06	49.35 ± 11.16	50.70 ± 9.84
	Echo	37.87 ± 10.23	43.32 ± 7.11	45.78 ± 9.15
t		2.266	2.880	2.319
*P*		0.026	0.005	0.023
LVEF (%)	MRI	60.31 ± 4.86	56.75 ± 7.74	56.57 ± 6.59
	Echo	56.65 ± 7.99	53.35 ± 6.63	51.62 ± 7.32
t		2.475	2.108	3.186
*P*		0.015	0.038	0.002

Note: LVEDV, left ventricular end-diastolic volume; LVESV, left ventricular end-systolic volume; LVEF, left ventricular ejection fraction; MRI, magnetic resonance imaging; Echo, echocardiogram. The t and *P* represent the difference of corresponding indexes between MRI and Echo groups

## Discussion

Although ANT represents a class of highly effective and broad-spectrum anticancer drugs that help improve patient survival and reduce tumor recurrence, they are also cardiotoxic and may impair myocardial function. Therefore, early and accurate assessment of cardiac function status has important clinical significance for patients receiving ANT chemotherapy [16]. Blaes et al. [17] investigated the effects of ANT on the myocardium of breast cancer and non-Hodgkin lymphoma patients after chemotherapy and found that hscTnT levels were significantly increased. Xu et al. [18] also reported that patients with gastric cancer showed a progressive increase in hscTnT after 3 and 6 cycles of ANT chemotherapy. In our study, there was no significant difference in serum hscTnT levels between cancer patients and healthy subjects before chemotherapy, and the serum hscTnT levels were significantly increased in patients with breast cancer and gastric cancer after 6-cycle of chemotherapy compared with the baseline value. These findings were basically consistent with the results of previous studies.

Currently, the techniques for assessing left ventricular function after chemotherapy in cancer patients include ECHO and cardiac MRI. ECHO is performed using color Doppler technology to evaluate heart function and heart structure, which can more carefully observe the size of the heart cavity, measure heart function, and observe the changes in the heart valve, including valve stenosis and valve insufficiency [10]. MRI is a non-invasive imaging technology that can observe various large blood vessels, pericdial disease and cardiomyopathy, such as primary cardiomyopathy, hypertension, aortic valve lesions, aneurysms, constrictive pericarditis and so on [12]. Previous studies [19–21] demonstrated that ECHO and cardiac MRI had equivalent values in the diagnosis of ventricular function. Liu et al. [14] found a good correlation and consistency between the two methods in terms of left ventricular systolic function evaluation. Using ECHO, Abdar et al. [22] found a decrease in peak early over peak late diastolic velocity (E/A) in patients after anthracycline use. However, Liu et al. [23] revealed that left ventricular end-diastolic and end-systolic diameters, LVEF, and E/A in breast cancer patients after T1 – T4 chemotherapy with ANT were not significantly different from those before chemotherapy.

Comparatively, we found that LVEDV, LVESV and LVEF measurements were lower in ECHO than in MRI, which was the same as Carly’s study [24]. In addition, the Bland-Altman diagrams were used to analyze the consistency of the results of the two methods and showed that the difference in LVEDV, LVESV and LVEF measured by ECHO and cardiac MRI in patients with breast and gastric cancer patients after chemotherapy was large, but the results of the two examination methods were in good agreement. Our findings were basically consistent with the above study results. Collectively, we compared and analyzed whether ANT resulted in different cardiotoxicity in patients with different types of tumors, and further verified the consistency of the assessment results of the left ventricular function obtained from ECHO and cardiac MRI.

Our study still has the following limitations. First, it was a single-center study with a relatively small sample size (including only 40 patients with breast cancer and 40 patients with gastric cancer), which might have affected the statistical power. Second, we only analyzed the left ventricular function in patients with breast and gastric cancer before and after 6 cycles of chemotherapy but lacked further validation compared with after other cycles of chemotherapy. Third, it should be noted that the cancer patients were included irrespective of their metastatic status and previous treatment (except for no previous treatment with ANT prior to this study), which might have affected the study results to a certain extent. However, in this preliminary study, all first-time ANT-treated patients were assessed to obtain a real-world representation of the general perspective of this regimen and agreement between MRI and ECHO. Also, sub-group analyses could not be performed for the different treatment stages due to the small number of cases assessed. Therefore, it is necessary to further expand the sample size and collect more data at different time points to strengthen the reliability of the experimental results.

## Conclusion

In conclusion, this study shows the potential cardiotoxic effects of anthracycline chemotherapy in breast and gastric cancer patients and highlights the importance of cardiac monitoring during treatment. Both ECHO and cardiac MRI can provide valuable information on cardiac function, with ECHO being more accessible, while MRI offers greater accuracy and comprehensive evaluation. The agreement between the two methods suggests that ECHO can be a useful tool for routine cardiac monitoring, while MRI may be more suitable for research or complex clinical situations. Further studies are needed to explore the long-term cardiac implications of anthracycline chemotherapy in cancer patients and validate our study findings.

## Data Availability

The data used to support the findings of this study are available from the corresponding author upon request.
